# Invasive Group A Streptococcal Infections, Israel

**DOI:** 10.3201/eid0804.010278

**Published:** 2002-04

**Authors:** Allon E. Moses, Sara Goldberg, Zinaida Korenman, Miriam Ravins, Emanuel Hanski, Mervyn Shapiro, the Israeli Group, for the Study of Infections Caused by Streptococcus pyogenes

**Affiliations:** * Hadassah University Medical Center, Jerusalem, Israel; † Hebrew University Medical School, Jerusalem, Israel; ‡ Ministry of Health Streptococcal Reference Laboratory, Jerusalem, Israel

**Keywords:** *Streptococcus pyogenes*, prospective study, population-based epidemiology, surveillance, invasive disease

## Abstract

We conducted a prospective, nationwide, population-based study of invasive group A streptococcal infections in Israel. We identified 409 patients (median age 27 years; range <1-92), for an annual incidence of 3.7/100,000 (11/100,000 in Jerusalem). The mortality rate was 5%. Bacteremia occurred in 125 cases (31%). The most common illnesses were soft-tissue infection (63%) and primary bacteremia (14%). Thirty percent of patients had no identifiable risk factors for infection. Eighty-seven percent of pharyngeal carriers had the same serotype as the index patient. M types included M3 (25%), M28 (10%), and M-nontypable (33%). A marked paucity of M1 serotype (1.2%) was detected. The results highlighted concentrated pockets of invasive disease in the Jewish orthodox community (annual incidence 16/100,000).

Group A streptococcus (GAS) causes human disease ranging from noninvasive infections such as pharyngitis or impetigo to life-threatening conditions such as bacteremia, necrotizing fasciitis (NF), and toxic-shock syndrome (TSS). Invasive GAS infections are thought to result from entry of bacteria through the skin, although often the site of entry cannot be determined. Since the mid-1980s, retrospective reviews of invasive GAS disease in different geographic areas have described an increase in deaths from these infections (*1**-**4*). These studies have also emphasized the changing nature of the population affected and have shown that young, healthy persons often have severe infections (*4*,*5*). This increased severity of invasive GAS infections has produced an augmented search for new virulence factors and host determinants that may amplify the potential of this organism for producing disease. Since GAS vaccines are being developed by several groups (*6*,*7*), baseline incidence data on severe GAS infections are needed. Information regarding the geographic distribution of M types will assist in directing vaccine development to prevalent strains. Prospective population-based studies provide an assessment of the true incidence of severe infection and are thus the preferred method for studying the epidemiology of disease. Few such studies of severe GAS infections have been performed (*5*,*8*–*11*); no previous studies have encompassed the epidemiology of an entire country.

We report the clinical characteristics of patients and bacterial attributes of GAS isolates from a 2-year, nationwide, prospective, population-based study to determine the incidence of invasive GAS diseases in Israel. In the greater Jerusalem area, we conducted an in-depth study to determine the prevalence of carriage of GAS in household contacts of index patients with invasive disease.

## Methods

We studied invasive GAS infections in Israel from January 1997 through December 1998. Collaboration between the study center in Jerusalem and 24 of the 25 acute-care hospitals in Israel was coordinated with the infectious diseases consultant or the director of the microbiology laboratory at each hospital. These hospitals serve approximately 95% of the Israeli population.

We provided each hospital with kits for transporting bacterial isolates and a questionnaire requesting the following demographic and clinical data for each case: age, sex, infection site, presence of hypotension, and signs of organ damage, including renal failure, adult respiratory distress syndrome, disseminated intravascular coagulation, or mental changes.

In a 1995 census from the Israeli Central Bureau of Statistics, the population of Israel was 5,548,000; 81% were Jews. Of the total population, 698,000 were children ≤5 years of age, 990,000 were 6-15 years of age, 2,550,000 were 16-45 years old, and 1,310,000 were persons >45 years of age. The median age of the population in Israel was 27.4 years (23.2 in the greater Jerusalem area).

In the greater Jerusalem area, the total population was 602,100; 421,200 were Jews, and 180,900 were Arabs. Among the Jewish population, 120,000 were Orthodox Jews, with an average of 5.5 members per family, compared with 2.9 in the nonorthodox Jewish population. Using age-adjusted rates for *Human herpesvirus 3 (HHV-3)* infection (*12*), we estimated that 4,100 cases of varicella occurred annually in children <10 years of age in the Jerusalem cohort.

Invasive disease was defined as the isolation of GAS from a normally sterile site such as blood, joint, or wound infection or from deep tissue retrieved during surgery. Isolates from the throat, ear, and eye were excluded. TSS was defined according to conventional criteria (*13*).

A study nurse (S.G) interviewed household contacts (defined as persons who lived in the same household as the index patient) within 3 days of obtaining a positive culture from an index patient who had been admitted to a hospital (one of three medical centers) serving the greater Jerusalem area. Information was obtained about antibiotic treatment and the presence of a recent throat or skin infection. Pharyngeal cultures were obtained from all household contacts.

Hospital laboratories identified the isolate as GAS by using a commercial latex agglutination kit. We sent all isolates to the Israel Ministry of Health Streptococcal Reference Laboratory for M- and T-typing and confirmatory GAS antigen typing. The presence of the genes encoding the two exotoxins A and C (*spe*A, *spe*C) was assessed by polymerase chain reaction (PCR) (*14*).

Statistical analysis was done by using the chi-square test for differences in proportions. A p value of <0.05 was regarded as significant.

## Results

### Clinical and Epidemiologic Characteristics

During the 2-year study period, 24 medical centers in Israel submitted 423 specimens. Of these, 14 isolates were not included in the study: 6 were found not to be GAS, and 8 were isolates from an eye or ear infection. Thus, 409 isolates were available for further study. The audit of case reporting showed a sensitivity that ranged from 30% to 70% among different institutions. However, for patients with bacteremia the average sensitivity of case reporting was 75%.

The annual incidence of invasive GAS infection in Israel was 3.7 per 100,000 population (Table 1). The median age was 27 years (range 1 week to 92 years). Incidence was highest in children <5 years and adults >45 years of age (Table 1). For bacteremia and TSS, the annual incidences in the national cohort (Table 1) were 1.1/100,000 and 0.25/100,000, respectively, with the highest incidence in persons ≥45 years of age (p<0.001). The case-fatality rate of the national cohort could not be assessed because deaths were underreported.

**Table 1:** The incidence of diseases from the national cohort, the Jerusalem cohort, the cohort of patients with bloodstream isolates, and the toxic-shock syndrome cohort, by age group, 1997–1998

Age group (years)	National cohort: no. of patients	Annual incidence^a^	Jerusalem: no. of patients	Annual incidence	Bacteremia: no. of patients	Annual incidence	TSS: no. of patients	Annual incidence
<5	88	6.3	38	19	26	1.86	5	0.36
6-15	53	2.68	16	6.8	11	0.56		
16-45	116	2.27	41	8	24	0.47	8	0.16
>45	109	4.16	38	15	62	2.37	15	0.57
Unknown	43				2			
Total	409	3.69	133	11	125	1.13	28	0.25
Median age (yr)	27		24		48		49	

^a^Annual incidence = cases per 100,000 population. TSS = Toxic-shock syndrome.

The disease entities associated with invasive GAS infection are summarized in Table 2. Of the 409 cases, 125 (31%) had bacteremia, which was considered to be primary (14%) if no source of infection was identified. The median age of bacteremic patients was 48 years, considerably higher than the median age (27 years, p<0.001) of all participants. Of 42 cases with cellulitis, 59% also had bacteremia. Of 14 cases of necrotizing fasciitis, 4 were bacteremic, and GAS was isolated from deep fascia in 10 others. Fourteen patients had pneumonia: in 5 patients GAS was isolated from blood, in 2 from pleural fluid, in 1 from lung tissue, and in the other 6 from sputum. Of 10 patients with burns, 7 were from one burn unit. Three of these seven comprised a single nosocomial outbreak.

**Table 2:** Clinical characteristics of invasive GAS infections: comparison of the national cohort to the Jerusalem cohort, 1997–1998

Disease	National cohort	%	Jerusalem cohort	%
Soft tissue infection^a^	272	67	88	66
Primary bacteremia	57	14	11	8.3
Pneumonia	14	3.4	4	3
Postpartum	10	2.4	6	4.5
Arthritis	8	2	1	< 1%
Lymphadenitis	6	1.5	2	1.5
Chickenpox	5	1.2	5	3.8
Meningitis	5	1.2	2	1.5
Peritonitis	5	1.2	4	3
PID	5	1.2	1	< 1%
Osteomyelitis	4	< 1%	2	1.5
Others	14	3.4	5	3.8
Unknown	4	< 1%		
Total	409	100	133	100

^a^Soft tissue infection includes 35 patients with abscesses, 14 with necrotizing fasciitis, and 10 with burns. PID = pelvic inflammatory disease.

In the national cohort, of 28 cases defined as TSS, 16 (57%) were male. In 20 (71%) of the TSS patients, GAS was isolated from blood cultures; in 10 this bacteremia was primary. Six had concurrent NF, two were children with chickenpox, and two had cellulitis. Only one patient was known to be positive for HIV infection.

### Characteristics of Bacterial Isolates

Serologic typing was performed for 401 isolates (98%). Of these, 33% were M-nontypable. The typable strains belonged to 23 different M serotypes. The most common strain was M3, constituting 25% of all isolates. The next most common serotypes were M28 (10%), M2 (5%), M62 (4%), M41 (3%), and M12 (3%). M1 was isolated from only five cases (1.2%). Four isolates gave a positive serologic reaction with two M serotypes.

Among the M-nontypable isolates, 21 different T serotypes were identified (data not shown). The most prevalent were T28 (11%), T12 (9%), T11 (8%), and T3/13/B3264 (8%); 28% were T-nontypable. T serotypes were different from those usually associated with M1. The M serotype distribution was similar in the national and Jerusalem cohorts.

Four hundred one isolates were tested for the presence of *spe*A or *spe*C by PCR. Ten percent were positive for *spe*A in both cohorts. Thirty-two percent and 37% were positive for *spe*C in the national and Jerusalem cohorts, respectively. Of the 101 M3 strains, 21% were positive for *speA.*

### Jerusalem Cohort

Of 409 patients who could be evaluated, 133 were from the Jerusalem area. In Jerusalem, the audit of case reporting showed a sensitivity of 94%. The median age was 24 years. The annual incidence of disease was 11/100,000 (Table 1). In this cohort the highest annual incidence was in the orthodox Jewish community (16/100,000; Table 3). The incidence differed by age group, with the groups <5 years (19/100,000) and >45 years (15/100,000) having the highest incidence (p < 0.01). Males accounted for 65%. In the Jerusalem group there were 86% Jews and 14% Arabs (Table 3).

**Table 3:** Religious distribution of patients in the Jerusalem cohort, 1997–1998

Religion^a^	Number (n=133)	%	Annual incidence^b^
Jewish	113	85	13
Orthodox Jewish	39/113	26	16
Moslem/Christian Arabs	18	14	5
Other	2	1	

^a^The Orthodox Jewish cohort is included in the Jewish cohort. ^b^Annual incidence = cases per 100,000 population.

The overall case-fatality rate in the Jerusalem area was 5% (7/133) but was 14% (6/44) among patients with bacteremia. Four of the seven patients who died in Jerusalem were >65 years of age.

All five cases of chickenpox-related GAS infection were in the Jerusalem cohort, for an estimated attack rate of 61/100,000 cases per year. One child died of the infection. All were children <4 years of age from orthodox Jewish families, but no epidemiologic association could be demonstrated since strains belonged to a variety of M serotypes.

In the Jerusalem cohort of 133 cases, we assessed the presence of underlying medical conditions that may predispose to GAS infection. Forty patients (30%) had no underlying disease. Nineteen percent had an acutely infected skin lesion. Twelve percent had a chronic skin condition, which was acutely infected with GAS. Ten percent of patients had diabetes mellitus, and nine percent had various forms of cancer. Nine pregnant women (7%) were infected before or shortly after delivery. Five children had chickenpox.

In the Jerusalem cohort, pharyngeal cultures were obtained from 302 contacts of 60 index patients. Relatives of 73 index patients were not studied because of lack of informed consent (*22*), nonavailability due to early discharge (*19*), or absence of family contacts (*32*). The mean number of family contacts per index patient was 5 (range 1-9). Twenty-eight index patients were associated with 61 household carriers of GAS, 75% of whom were ≤15 years old. Only one contact (a child with pharyngitis) was symptomatic. The gender distribution of the carrier cohort was similar to that of the general population. Comparison of the M serotypes of the carriers and their respective index patients disclosed identity in 87% of cases.

## Discussion

Our prospective population-based study is a first nationwide survey of the incidence of invasive GAS. The annual incidence of invasive GAS infections in Israel (3.7/100,000) is similar to that reported from Pima County, Arizona (*4*), and Atlanta, Georgia (*9*)*,* but is considerably higher than that reported for Ontario, Canada (1.5/100,000) (*8*). The annual incidence in the Jerusalem cohort was three times higher (11/100,000) than that of the national cohort, reflecting, at least in part, a greater accuracy of reporting, achieved by frequent contact of the study nurse with the three medical centers. The relatively large number of orthodox Jews living in the Jerusalem area may also have contributed to the higher incidence of infection in this city. Unlike Ontario or Connecticut (*8*,*11*), reporting invasive GAS infections in Israel is not mandatory, contributing to the relatively low reporting accuracy. Thus, the true incidence of invasive GAS disease in Israel may be closer to that of the Jerusalem cohort, which is significantly higher (p<0.0001) than that reported by other population-based studies. We made routine telephone calls to the hospital contacts to encourage participation and confirm that cases were being reported. Audits of one third of the large and small hospital laboratories were conducted twice during the study period to evaluate the proportion of cases actually reported. Since some of the Arab population in Jerusalem uses the East Jerusalem Arab hospitals, which were not included in the study, infections occurring in a small proportion of Jerusalem Arab patients may have been missed.

Risk factors for GAS infection were not studied in the national cohort. In the Jerusalem cohort, 30% were previously healthy patients without evident risk factors. This finding is consistent with those of other studies, supporting the notion that underlying illnesses appear to play an important role in the occurrence of invasive GAS disease (*4*,*8*,*9*,*15*–*17*). Previous skin lesions, diabetes mellitus, and cancer were the most common conditions predisposing to GAS infection. Alcoholism and AIDS are relatively rare in Israel (*18*) and were not found to be risk factors for our patients.

In Israel the incidence of invasive streptococcal disease in children was higher than reported previously (*9*), consistent with our earlier finding that children with GAS bacteremia in Jerusalem were younger than those reported by others (*19*). In Jerusalem the incidence of GAS infection in the <5-year age group was higher than previously reported (19/100,000). This incidence remained elevated (14/100,000) even after five cases of an infection secondary to chickenpox were excluded from analysis.

In Jerusalem, 40% of children (≤15 years of age) with invasive GAS infections were from the orthodox Jewish community. The overall incidence for this group was 16/100,000 population and was probably even higher in children ≤15 years of age, although data for age distribution were not available for this group. In this community, families are large and the relatively crowded living conditions may facilitate the spread of streptococci (*20*). Recently, a higher pharyngeal carriage rate (odds ratio 5.0; 95% confidence interval 2.1-11.9) of GAS was reported for an orthodox Jewish community in London (*21*). In Jerusalem, the incidence of GAS infection was also much higher in the group >45 years of age (Table 1). Thus, the incidence of severe GAS infections reported in the Jerusalem area is much higher than previously reported. The mortality rate (5%) in the Jerusalem cohort was lower than that reported by Davies et al. (*8*) and Zurawski et al. (9) but is similar to the rate reported for bacteremic patients at the Hadassah Medical Center in Jerusalem (*22*).

Both bacteremia and TSS occurred at a substantially older age (median age 48 and 49 years, respectively) than the median age of the general population in Israel (Table 1). The annual rate of TSS in our study was similar to that reported by Davies et al. (0.2/100,000), who also found that severe disease occurred preferentially in older patients. NF was relatively rare in Israel (annual incidence 0.1/100,000), accounting for 3.4% of all patients and 4.5% of the Jerusalem cohort patients. This was similar to the percentage reported by Zurawski (3%) but considerably less than that reported by Davies et al. (13%). Zurawski et al. (*9*) suggested that the low incidence of NF might have been due to ascertainment bias, engendered by the laboratory-based study methods, which may have missed cases of NF without concomitant bacteremia. However, in Jerusalem the close association between the study team and their clinical and laboratory counterparts in all three medical centers makes such an ascertainment bias unlikely. The differences between population-based studies may be due to microbiologic attributes of the strains involved or other unknown factors.

Nosocomially acquired invasive streptococcal infections were relatively rare. Postpartum infections accounted for 2.4% and 4.5% of the national and Jerusalem cohorts, respectively. Although this infection can be hospital acquired (*22*,*23*), GAS may also arise from the patient’s own bacterial flora (*8*). Seven of the 10 burn-related infections occurred in one hospital, and three cases belonged to a single serotype (M3, *spe*C+). Such an outbreak has been reported to occur by transfer of GAS from medical personnel to patients (*24*–*29*).

Our data support the assumption that chickenpox is a risk factor for invasive GAS disease (*8*,*9*,*30*–*33*). In our cohort, all children with chickenpox were from orthodox Jewish families. Nevertheless, cases were caused by diverse M serotypes and occurred in several different city neighborhoods without any epidemiologic link between them.

The most striking microbiologic characteristic of the GAS isolates of both the national and Jerusalem cohorts is the paucity of the M1 serotype. This finding contrasts with many previous reports, which described the M1 serotype as the prominent isolate from patients with invasive GAS infection, particularly those with NF or TSS (*34*–*40*). Our M-nontypable strains had 21 distinct T serotypes that differed from those usually associated with M1 strains. Thus, *emm* typing would be unlikely to categorize these nontypable isolates as M type 1.

The association between M1 and the production of exotoxin A is well established (*37*) but not universal (*41*). The prevalence of *spe*A in our cohort (10%) was lower than that found by Zurawski (34%) (*9*) and Kiska, who observed that 98% of M1 and M3 outbreak strains were *speA* positive (*5*). Half our *speA*-positive cases were serotype M3, but few were associated with TSS and NF. We concur with Davies (*8*) that factors other than *spe*A play a role in TSS pathogenesis. These findings and the paucity of the M1 serotype among our isolates suggest that no single invasive clone is responsible for severe disease (*20*) and that strains attain their virulence through means other than *spe*A. The relative prevalence of *spe*C in our study was similar to that found by others (*8*).

Serotype M3, the most frequent M type in our cohort and a relatively common isolate in other studies of invasive GAS (*41*,*42*), seems to have replaced M1 as the leading cause of invasive streptococcal disease (*8*). In a retrospective analysis of GAS bacteremia in Jerusalem over a 6-year period (1987-1992), none of the 41 isolates available were serotype M3, 11 (27%) were nontypable, 4 were M12, and 2 were M1 (*22*). Thus, an unexplained increase in the rate of invasive M3 strains in Jerusalem has occurred. Nevertheless, this increase has not been accompanied by a change in the absolute number of bacteremic streptococcal cases per year, and the mortality rate has remained constant. Therefore, we cannot conclude that infections with M3 result in more cases of bacteremia or are more virulent. Only 20% of our M3 strains harbored the *spe*A gene, compared with 100% of the strains from Japan (*41*). In Israel, 67% of 21,517 GAS isolates (mostly from pharyngeal swabs taken over a 10-year period) were M-nontypable, and 99% were T-typable (*43*). The most prevalent M serotypes were M12 (17%) and M1 (6%), and the most prevalent T type was 3/13/B3264 (20%). Ten years later, Yagupsky et al. found 90% strains (10/13 cases) of GAS isolated from children with bacteremia to be M-nontypable (*44*).

The M28 serotype, accounting for 10% of our cases, was reported to be a common serotype in invasive GAS diseases by some investigators (*8*) but not by others (*9*). We also had a relatively high percentage of M-nontypable isolates. This finding is in contrast to those who have been able to serologically type >90% of isolates (*41*) but is similar to findings of surveys in which 60% or 80% of isolates were nontypable (*9*,*45*). *emm*-typing (*46*) may clarify the actual M type of those strains.

In the Jerusalem cohort, we found a particularly high prevalence of patients with family members who had GAS in their pharynx. As reported previously (*47*), the M types of almost all isolates (87%) from household contacts were identical to those found in the index patients. We chose to administer preventive antimicrobial therapy to positive contacts, although this practice is still controversial (*48*). None of the contacts had invasive disease.

As in other studies (*47*), we found that asymptomatic carriers were mostly young children. Whether the index patients were infected from an asymptomatic carrier or vice versa is impossible to determine. The reasons for one person remaining an asymptomatic carrier while another has a severe, sometimes lethal infection have not been clarified. Whether there are any genetic differences between the index patient/contact pair of bacterial isolates or whether varying virulence genes of GAS are expressed under different clinical conditions remains to be determined. Further studies of GAS epidemiology and pathogenesis are required to determine the reasons for acquiring severe invasive GAS diseases in specific hosts. This knowledge will allow a more accurate definition of the risk factors for these infections and may lead to development of effective intervention strategies.
